# Tell me who's your neighbour and I'll tell you how much time you've got: The spatiotemporal consequences of residential segregation

**DOI:** 10.1002/psp.2561

**Published:** 2022-03-28

**Authors:** Boróka B. Bó, Denys Dukhovnov

**Affiliations:** ^1^ University of Essex Colchester UK; ^2^ University of California Berkeley California USA

**Keywords:** ethnic segregation, neighbourhoods, spatial econometric models, sociotemporal inequality

## Abstract

Relying on data from the United States Census and the American Time Use Survey (2010–2017), we examine how residential segregation influences per capita discretionary time availability in Los Angeles, New York City and Miami. We find a sizable disadvantage of being Latinx for discretionary time availability. Non‐Latinx Whites have 182 extra hours of per capita discretionary time per year than do Latinx individuals. Both within‐neighbourhood and adjacent‐neighbourhood influences matter. In most neighbourhoods, segregation is correlated with having more discretionary time. Individuals in highly segregated areas have approximately 80 more hours of discretionary time per year than those living in diverse areas. This suggests that in addition to socioeconomic, cultural and well‐being benefits, ethnic enclaves may also impart temporal advantages. However, we find that there may be diminishing marginal returns with increasing segregation in surrounding areas. Sociodemographic characteristics explain over one‐quarter of the variance between segregation and discretionary time availability.

## 1 INTRODUCTION

Social scientists have studied the relationships between ethnic residential segregation and socioeconomic inequality for over 100 years (DuBois, 1899; Massey & Denton, 1988). We continue to unpack how inequality clusters spatially, contributing to the intergenerational transmission of disadvantage. Yet, despite our calls for action, the United States is still a highly ethnically segregated society. For example, although the Latinx population is projected to double in the next 40 years—forming one‐third of the total population of the country and the largest ethnic minority group in the United States—their population growth has been accompanied by increased segregation from other ethnic groups (Bernstein, 2013; Lichter et al., 2015; Rugh & Massey, 2014). These rapidly shifting patterns in Latinx geographic segregation point to widening economic, cultural and social distance from other ethnic groups (Rumbaut, 2011; Tienda & Fuentes, 2014). Recent research has also found that residential segregation has negative economic effects for this population, likely influencing their population‐level time use profiles (Bernstein, 2013; De la Roca et al., 2018; Hamermesh, 2019). This has both micro‐ and macro‐level consequences: segregation shapes individual life chances, influences local opportunity structures and the characteristics of neighbourhoods (Alba & Foner, 2015, Crowder & South, 2008).

This study seeks to reorient current theorizing on Latinx residential segregation by centering sociotemporal inequalities. We introduce the idea that it is imperative to consider time when theorizing about how ethnic residential segregation affects needed resources. Focusing on per capita discretionary time left over after meeting daily survival needs, we examine the puzzle of ethnic residential segregation and discretionary time availability. Though we know that neighbourhood‐level sociodemographic characteristics are central for the social processes that drive stratification (Castañeda et al., 2015; Harding, 2007), the spatial distribution of time availability is still understudied (Castañeda, 2018). Methodological complexities and data limitations have thus far prevented us from concretely examining how ethnic segregation shapes time use disparities (Castañeda, 2018). This is unfortunate, since discretionary time is necessary for combating entrenched inequalities, garnering socioeconomic resources, and for all aspects of individual and community well‐being (Giurge et al., 2020; Goodin et al., 2008; Kalenkoski & Hamrick, 2013; Massey & Fischer, 2000; Williams et al., 2016).

Furthermore, neighbourhoods are not isolated islands. They are influenced by each other: neighbourhood mobility and sociodemographic patterns are altered by the ethnic compositions of adjacent neighbourhoods (Crowder & South, 2008; Wilson & Taub, 2007). Yet, we still do not know how ethnic segregation in adjacent neighbourhoods may shape discretionary time availability in a nearby neighbourhood. Ethnographic research suggests that segregation matters for between‐neighbourhood time use, influencing the well‐being of residents (e.g., commuting patterns and waiting times) (Castañeda, 2018; Edwards, 2017). However, until now, we have been unable to examine this systematically via large‐scale quantitative data. Thus, our understanding is incomplete when it comes to how the interplay between ethnic segregation and neighbourhood‐level sociodemographic conditions shapes discretionary time availability.

We begin by putting prevalent theoretical perspectives from the neighbourhood effects literature in conversation with theories on segregation and from the sociology of time, explicitly touching on the hypotheses undergirding our study. Next, we provide an overview of the data, followed by our downscaling method producing local estimates of discretionary time, then review and compute our segregation measures. We ask three related questions:

1. Does per capita discretionary time availability vary spatially, and if so, does residential ethnic segregation matter for discretionary time availability?
2. How do sociodemographic characteristics influence the above?
3. Do the characteristics of adjacent neighbourhoods affect the relationship between segregation and time availability?

## 2 DISCRETIONARY TIME AND NEIGHBOURHOOD EFFECTS

While no one has more than 24 h in a day, not everyone has the same amount of discretionary time. We offer a more precise operationalization of discretionary time in our methods section, but the construct can be efficiently summarized as the time left over for discretionary activities after the minimal amount of time needed to satisfy bodily, financial and household needs has been spent. Discretionary time is imperative for individual well‐being and for the maintenance of a functioning society (Goodin et al., 2008; Hamermesh, 2019; Rosa, 2013; Whillans, 2019; Williams et al., 2016). A growing body of literature notes that contextual factors and sociodemographic variables influence discretionary time availability (Giurge et al., 2020; Hamermesh, 2019; Kalenkoski & Hamrick, 2013). Overall, those with more economic and cultural resources have more control over their time and more autonomy over their discretionary time. Those with less resources spend extra time to make enough money. Those with higher status can purchase time by outsourcing menial tasks (Hamermesh, 2019). Thus, discretionary time is intimately intertwined with social inequalities. Per capita discretionary time availability is a highly responsive measure to sociodemographic constraints and opportunities, while also being sensitive to historical and structural conditions: when structural inequality impedes on discretionary time availability, this has both individual and societal ripple effects (Goodin et al., 2008; Hamermesh, 2019; Williams et al., 2016).

### 2.1 Ethnic residential segregation and discretionary time availability

Residential segregation refers to the sorting of large, homogeneous groups of people into specific spatial concentrations (DuBois, 1899; Massey & Denton, 1988). Explanations as to how residential segregation affects the lives of the Latinx population in the United States can be roughly grouped into two categories: studies looking at the detrimental effects or those noting potential benefits. Existing literature has not explicitly examined how ethnic segregation may be detrimental for discretionary time availability, but it does point to multiple key propositions.

Residential segregation may undermine the socioeconomic outcomes of the Latinx population, by constraining them to live in neighbourhoods with less public investment, higher poverty, underemployment and unemployment, underfunded institutions and limited access to stable jobs (Borjas, 1994; Castañeda, 2018; Edwards, 2017). Socioeconomic status (often measured by income and education attainment levels) may force individuals to live in more impoverished neighbourhoods. These neighbourhoods have less stable, owner‐occupied housing, higher population density and high levels of ethnic segregation (Alba & Foner, 2015; Castañeda, 2018). The above could lead to a higher likelihood of segregation being detrimental for per capita discretionary time availability, as individuals may need to spend extra time to mitigate the effects of neighbourhood instability by commuting longer distances and working multiple jobs (Castañeda, 2018; Cutler et al., 2008; Edin et al., 2003; Giurge et al., 2020; Holzer, 1991).

Lower‐income, segregated neighbourhoods also tend to experience disproportionately higher rates of disruption (Sharkey et al., 2014). These can range from lower high school completion rates due to familial stressors to higher rates of single women‐led households. In such situations, extended family members may need to take on the responsibility of caring for dependents at the expense of their per capita discretionary time availability (Feldmeyer, 2010; Shihadeh & Barranco, 2010). Thus, ethnic residential segregation may further exacerbate already‐present gender differences in free time (Bianchi et al., 2000, Castañeda, 2018). Women living in segregated neighbourhoods may need to take on extra jobs to support their families, cutting into their discretionary time availability at home. Women may also feel more obligated to perform gendered labour for extended family nearby (Pinto & Ortiz, 2018).

However, research on ethnic enclaves has suggested that in some circumstances, ethnic segregation may be beneficial. The presence of extended kinship networks in ethnically segregated communities could potentially distribute economic risks (Menjivar, 1997). People choosing to live in communities where their ethnicity is over‐represented may tap into social networks more efficiently. These could include job connections, carpool networks and emotional support, reducing the likelihood of family instability (Fomby et al., 2010). This could increase discretionary time availability as community members could receive time and money‐saving assistance. Residential segregation could also contribute to community well‐being by offering eldercare, childcare and market labour (Waldfogel, 1999). Through the above mechanisms, residential ethnic segregation can create self‐contained markets for ethnic goods and access to coethnic sources of capital, leading to higher median income levels, lower unemployment rates and higher rates of owner‐occupied housing (Cutler et al., 2008; Edin et al., 2003; Fischer & Massey, 2000; Menjivar, 1997). This could have positive consequences for discretionary time availability.

Ethnically segregated neighbourhoods may also increase discretionary time availability in more tangible ways. The ability to speak a common language makes a concrete difference when it comes to efficiently navigating one's environment. When norms and customs are familiar to all and communities can establish ethnic institutions (churches, stores, etc.), common economic and well‐being goals are accomplished more efficiently (Fine, 2002; Lazear, 1999). This is particularly beneficial for lower SES individuals (Edin et al., 2003). Thus, residential segregation could reduce the chance of lacking needed discretionary time.

## 3 ADJACENT NEIGHBOURHOOD EFFECTS AND DISCRETIONARY TIME AVAILABILITY

While multiple researchers have explored how segregation may matter for neighbourhood‐level infrastructure and resources (Crowder & South, 2008; Denton & Massey, 1991), it is unclear how segregation in adjacent neighbourhoods may influence discretionary time availability in an area. This is imperative to consider, as existing research shows that neighbourhoods influence each other, with residential segregation having important adjacent‐neighbourhood consequences (Castañeda, 2018). This literature can be sorted into two broad categories: studies looking at spillover effects and those focusing on containment. When we refer to spillover effects, we mean that the characteristics of one neighbourhood may influence those of an adjacent neighbourhood. Studies on the spillover effects of segregation have found that both proximity and population size matter. The proximity of a neighbourhood to one with a highly segregated large minority population strongly predicts the likelihood of future change in local neighbourhood characteristics (Denton & Massey, 1991). This can ensue from ‘white flight’, resulting in minority population members moving into an adjacent neighbourhood previously inaccessible to them (Crowder & South, 2008). Ethnically segregated areas can also be influenced by conditions prevalent in the neighbourhoods adjacent to them. For example, if group‐level inequality is high between two neighbourhoods, these conditions may contribute to housing instability, displacement, and the necessity to dedicate needed discretionary time to labour, just to be able to afford continuing to live in one's own neighbourhood (Ocejo, 2011; Policy Link, 2016).

To understand how ethnic segregation in adjacent neighbourhoods could spill into the time use profile of another, we need to consider that time and money are distinct while being intimately intertwined (Sampson et al., 1999). For example, an economically well‐off, less segregated locality may ‘buy time’ via care or household labour from a nearby economically less well‐off, potentially segregated neighbourhood. This would lead to the necessity for those living in the segregated, less economically privileged area to commute to other neighbourhoods for work, influencing their discretionary time.

Adjacent neighbourhood characteristics could also have the opposite effects on local characteristics. They may curtail the tendency for one population to interact with adjoining populations, containing population and neighbourhood‐characteristics to a particular area (Lee, 1966; South & Crowder, 1997). When it comes to the containment of discretionary time availability to neighbourhoods, we can think of this as such: The concentration of an ethnically segregated, possibly impoverished group to a neighbourhood may lead members of nearby more economically well‐off, perhaps less ethnically segregated neighbourhood to avoid the area. This will lead to the entrenchment of both economic and discretionary time scarcity in the area avoided, as the likelihood of economic investment and job creation decreases (Lee, 1966; South & Crowder, 1997). Here, individuals may need to work harder to survive: juggling multiple low‐paying jobs, while navigating crumbling infrastructure and institutions. Their efforts will influence their per capita discretionary time availability.

The ethnic composition of adjacent neighbourhoods may also affect the conditions of a local neighbourhood (Crowder & South, 2008). In the case of discretionary time availability being contained, this could happen through ethnic segregation undermining between‐neighbourhood time exchanges. Or conversely, an ethnically segregated neighbourhood may be more socially cohesive, which could contain discretionary time availability to the area. In this scenario, members of segregated yet cohesive neighbourhoods would be less likely to form new social ties (which require time to establish) in adjacent neighbourhoods (DiPrete et al., 2011). As the literature above illustrates, maintaining focus on discretionary time availability is imperative for a better understanding of the repercussions of ethnic segregation.

## 4 RESIDENTIAL SEGREGATION AND INEQUALITY

Ethnic residential segregation is consequential for multiple reasons: it influences how individuals navigate and experience neighbourhoods, undergirds the stability of social networks, shapes health outcomes, access to resources and interactions with institutions (Bayer et al., 2008; Durlauf, 2004; Massey, 2001). The above consequences all have the potential to influence inequalities in discretionary time availability. In lieu of outlining the extensive body of existing literature on residential segregation, we review aspects relevant to our study.

The existing literature is in disaccord as to whether segregation is harmful or beneficial for the Latinx population when it comes to their noneconomic outcomes. Some argue that segregation for them may be beneficial (network cohesion, access to fresh vegetables, etc.), while others assert that as the segregated Latinx tend to be disproportionately exposed to neighbourhood disadvantage, this carries detrimental health consequences (Do et al., 2017). This presents a puzzle: Latinx ethnic residential segregation may have different consequences, depending on how it shapes the noneconomic outcome of discretionary time. If ethnic residential segregation is beneficial for discretionary time availability, it may be beneficial for population‐level mental and physical well‐being. However, if ethnic residential segregation is detrimental for time availability, the segregated group may be doubly disadvantaged (Giurge et al., 2020; Rumbaut, 2011).

There are several reasons to suspect that ethnic segregation might have an independent effect on per capita discretionary time. Starting with Durkheim, multiple researchers have argued that distinct groups experience and share time differently (Durkheim & Swain, 2008; Gell, 1992). Segregation creates self‐contained social groups, curtailing a group's ability to meaningfully interact with others outside of their segregated social networks, limiting between‐group transmission of time and temporal perspectives (Bourdieu, 1964; Echenique & Fryer, 2007; Massey & Denton, 1988). Notwithstanding the extensive literature on the topic, pressing gaps remain. Despite knowing that economic factors are salient, and that discretionary time is as important for individual and societal well‐being as money is, we do not know how structural conditions—such as ethnic segregation—influence the necessary resource of discretionary time. While we know that individual‐level sociodemographic variable shape time availability, how neighbourhood‐level sociodemographic characteristics—such as population density and housing type, group‐level income inequality, median income, unemployment rates, area‐level education attainment and household composition—may influence the relationship between segregation and per capita discretionary time is still uncharted. Similarly, though the existing literature has noted that neighbourhoods influence each other (Crowder & South, 2008; Wilson & Taub, 2007), it is unclear how segregation in adjacent neighbourhoods, along with their socioeconomic environment, may predict discretionary time availability at a given location.

To study the relationship between ethnic segregation and per capita discretionary time, we use data from three highly segregated localities in the United States when it comes to Latinx segregation: New York City, Miami and Los Angeles (Alba & Foner, 2015). Many areas in these cities rank high on the Information Theory Index, an entropy‐based measure of diversity capturing how segregation varies between neighbourhoods (Massey & Denton, 1989; Reardon & Firebaugh, 2002). Specifically, we use the above cities' labour market commuting zones, as they delineate local experiences better (as counties reflect political borders)^1^. Though highly segregated, these localities also contain integrated areas. Further, our labour market commuting zones house dissimilar Latinx groups, allowing us to honour the cultural heterogeneity of the population. The Los Angeles region contains individuals with mainly Mexican and Salvadoran origins. Caribbean‐origin Latinx are prevalent in the New York City area, whereas many with Cuban origins live in the Miami region (Alba & Foner, 2015). These localities serve as great case studies for the examination of the linkages between Latinx residential segregation and per capita discretionary time availability.

## 5 DATA

Our analyses rest on two data sources. The first consists of 8 consecutive years of the ATUS (2010–2017, *N* ≈ 96,000). This is a nationally representative, diary‐based survey of American adults, recording the amount of time spent on various activities, where, and with whom. The annual survey sample is drawn from the participants of the Current Population Survey (CPS), conducted by the U.S. Census Bureau. A crucial feature of the survey is geographically linking respondents to their county of residence. This link exists for 10% of counties, encompassing the most populous suburban and urban neighbourhoods. This feature undergirds our methods of choice and the scope of our analyses.

Our second data source is the 2010 U.S. Census county and census block TIGER shape files—with corresponding population attributes by age, sex, race and ethnicity—assembled by the National Historical Geographic Information System (NHGIS) (Manson et al., 2017). This enables us to scale down the time use rates to the specific demographic groups (considering age, sex and ethnicity) living in the subcounty local areas, and to calculate our segregation indexes. Both datasets allow individuals to self‐select into our Latinx group if ‘they are Hispanic *or* Latino, of any race’.^2^

## 6 METHODS

### 6.1 Discretionary time availability by demographic group

Our analysis centers three highly ethnically segregated localities in the United States: Los Angeles, CA (LA), Miami, FL and the New York City, NY (NYC). Since the CPS only has geographic links for respondents in populated urban and suburban counties, this precludes us from including the rest the country. Following precedent, we define discretionary time as time dedicated to leisure, socializing, religious activities and exercise (Goodin et al., 2008; Williams et al., 2016). For each city, we consider 12 subgroups: two ethnicity categories (Latinx and non‐Latinx White), two sexes (female and male), and three age groups (30–44, 45–54 and 55–69). The age groups capture seminal stages of working adulthood: early middle age, middle age and early late adulthood (Medley, 1980). Our choice was also influenced by sample size constraints, as the introduction of more detailed breakdowns would have led to dwindling sample sizes for select subgroups. This allows us to generate stable estimates for our 12 groups. Next, we introduce our downscaling method, using ATUS county‐level measures to approximate discretionary time for standardized, small areas.

First, we create a standard unit to which county‐level estimates of discretionary time will be downscaled. We downscale ATUS‐derived rates to 1‐km‐wide hexagonal grids. These hexagonal grids serve as the unit of analysis in our models. Our choice was guided by precedent, heeding the call to shift focus from census tracts as proxies for neighbourhoods, to more theoretically meaningful, standardized, comparable units of analysis (Chaix et al., 2005; Riley, 2018). Such continuous spatial surfaces have been used to characterize neighbourhood contexts in work examining the geographic distribution of resources (Chaix et al., 2005). Sensitivity analyses also show that 1‐km‐sized grids exhibit the lowest root mean squared error, producing the most accurate population characteristics (Lloyd et al., 2017). This is relevant, as census tract sizes can vary considerably within a county. Our uniform‐sized grids correct for this, allowing for accurate comparisons between areas. This scale does not require additional statistical techniques to regularize the data for comparability (Le Bras, 2008). As an added advantage, hexagonal grids correspond to coastal regions better than square grid cells, which is relevant given that our cities have extensive coastal areas. In sum, for our purposes, grids are superior alternatives to irregular geography (such as census tracts or block groups) (Lloyd et al., 2017).

Next, we fill the standardized hexagonal grids with our populations of interest. We spatially overlay the census blocks along with their population counts by ethnicity, age and sex on top of our grid lattice. This distributes the census block information to the overlapping grid cells in proportion to the block area falling within any such cell^3^. For instance, if block A has 1000 people of a certain demographic group, and block B has 2000, then a cell containing 50% of the area of block A and 75% of the area of block B will have 1000×0.5+2000×0.75=500+1500=2000 such residents. One assumption that underlies this process is that the population is distributed homogeneously throughout a census block. This a reasonable assumption, given that our 1‐km‐wide hexagonal grids are comparable to the population size of census tracts in places with high‐to‐moderate population density. Most urban blocks are quite small, generally containing housing of similar density and type, minimizing error.

Third, we generate county‐level estimates of discretionary time, employing recommended ATUS methodology (Bureau of Labour Statistics, 2020, p. 37).

(1) $$\bar{T}\;=\sum_{k}\frac{\sum_{i}\left[t_{\mathrm{ik}}\times W_{k}\right]}{W_{k}}.$$

Equation 1 shows this, where $\bar{T}$ is the average time estimate for a specific group of individuals (e.g., Latinx females, 30–44 years old), and t is the average daily time spent on a unique activity i in the data set by each respondent k, while $W_{k}$ is the respondent's sampling weight.^4^

Fourth, we multiply the cells with population attributes from step 2 with the county average rates of discretionary time by demographic group from step 3. This yields the total amount of discretionary time that members of each of the 12 population groups have in any given cell. In doing so, we must assume homogeneity in time use rates. This is a necessary assumption, as it allows us to calculate the weighted mean of discretionary time availability at the 1‐km grid level, further permitting meaningful, small‐scale comparisons^5^.

Fifth, we compute the population‐weighted average of discretionary time within each cell for each ethnicity. Finally, to refine our estimates, we propose a smoothing method to mitigate the side effect of arbitrary county cross‐border fall of in discretionary time values. This phenomenon is expected, as county‐level discretionary time rates are fitted to the underlying populations. Grid cells at the border of two counties may exhibit sizable differences between estimates of discretionary time for the same demographic groups. As one would not expect to find sharp transitions due to an administrative boundary, we ensure the estimates in our grids retain high degree of fidelity to the parent county rates generated from ATUS (step 3). The method reduces the intensity of cross‐border fall off as a function of the inverse‐distance of a cell from the county border (e.g., cells farther away are smoothed less), size of a county itself, and smoothing‐resistance‐factor dictated by the proportion of a cell population that is urban, since survey estimates are produced by the concentrations of people living in an area, and not its land.

**Figure 1:**
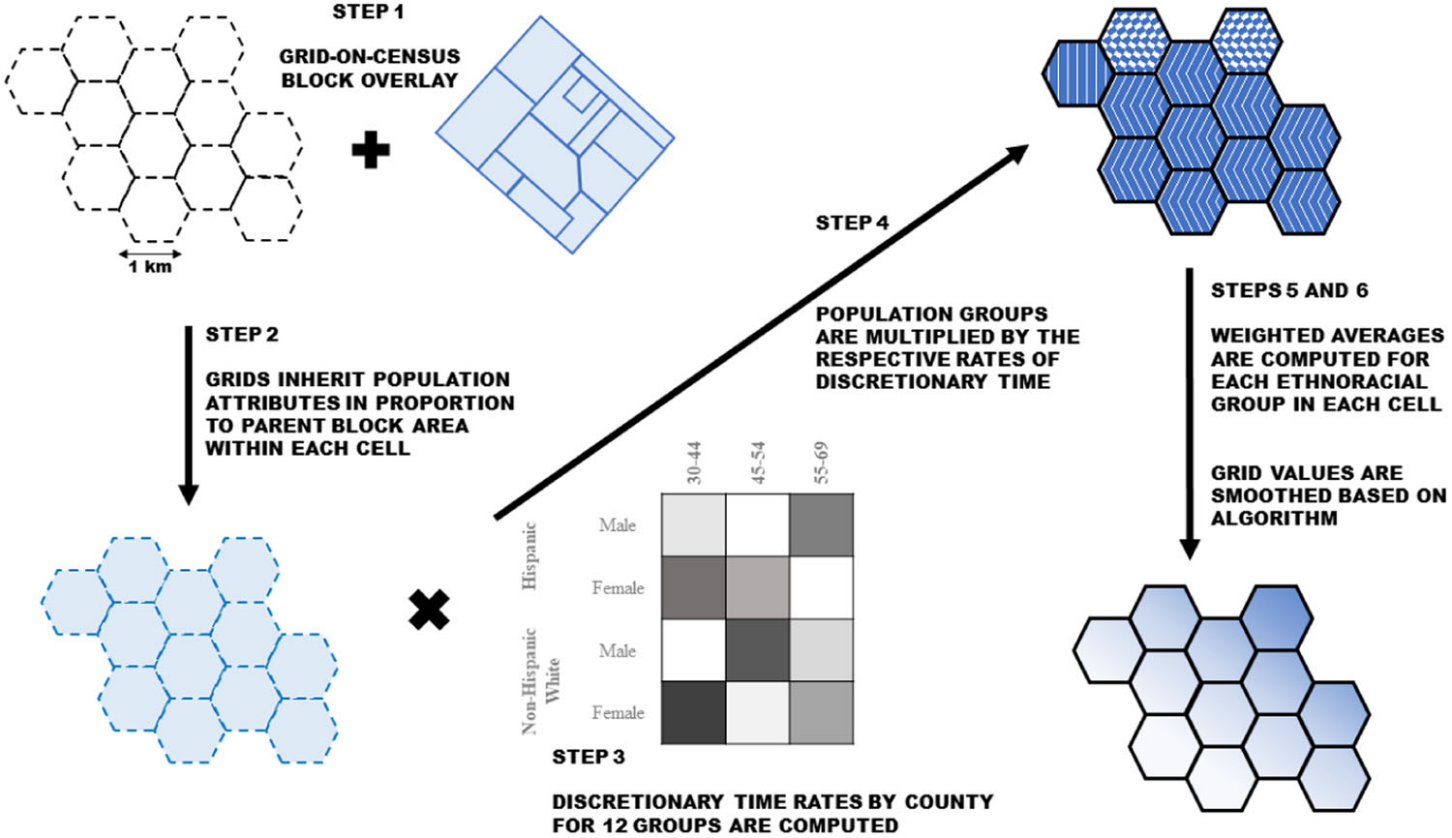
Downscaling and spatial adjustment procedures

Figure 1 provides a summary of our downscaling and spatial adjustment method.

### 6.2 Ethnic segregation

To measure ethnic segregation, we rely on two widely applied measures of unevenness and inequality. First, we adopt a measure of inequality developed by Roberto (2015), the Divergence Index (Roberto's D). To ensure that our results are comparable with much of the literature on residential segregation, we also use the Information Theory Index (*H*), describing the relative diversity or homogeneity in an area (Reardon & Firebaugh, 2002). Although both measures are area‐decomposable, allowing for multi‐group score calculations, we are interested in Latinx‐White segregation. Thus, we calculate two‐group D and H indices, for Latinx and non‐Latinx Whites. The *H* index is particularly fitting for our analysis, as it compares the degree of evenness in one group relative to the population of both groups (Massey & Denton, 1989). The U.S. Census Bureau also uses the *H* index to measure how large, homogeneous groups of people are sorted into specific spatial concentrations (Massey & Denton, 1988).^6^ Alternatively, the Divergence Index indicates how ‘unusual’ is the entropy of an observed value, relative to the theoretical/expected value (Roberto, 2015), reflecting segregation as the over‐ or under‐representation of population groups in a local area. Both indices are robust to the size of the underlying population, allowing for the comparison of various areas.

The *H* index is top‐bounded by the maximum value of 1, denoting maximum relative homogeneity. Although the lower bound is typically 0, the minimum possible value can be negative, representing relative ‘hyper‐integration’ or a complete mixing of groups within an area. The Divergence Index is strictly bounded between 0 and 1, denoting maximum integration and maximum segregation. To achieve the greatest possible accuracy in our D and *H* indexes, we use census blocks as underlying geographic units to proportionally allocate the population to the cells that contain them. Following Reardon and Firebaugh (2002), we first compute individual‐cell entropy scores

(2) $$E_{i}=\sum_{g=1}^{G}\pi_{g}\log\frac{1}{\pi_{g}},$$

where $E_{i}$ is the entropy score of a cell i within metropolitan region, and $\pi_{g}$ is the proportion of group g (Latinx or non‐Latinx White) in the cell. To obtain the Information Theory Index for each cell, we compute the difference between the cell and regional level entropy scores, standardized by regional entropy.

(3) $$H_{i}=\sum_{i=1}^{N}\frac{\left(E-E_{i}\right)}{E}.$$

Following Roberto (2015), the Divergence index for a cell is calculated as:

(4) $$D_{i}=\sum_{g=1}^{G}\pi_{\mathrm{ig}}\log\frac{\pi_{\mathrm{ig}}}{\pi_{g}},$$

where $\pi_{\mathrm{ig}}$ is the proportion of group g population in cell i and $\pi_{g}$ is the proportion of group *g* in the overall population.

### 6.3 Spatial modelling

Since we are interested in predicting the gap on average discretionary time availability between two populations, while accounting for local clustering or spillover effects, we rely on spatial econometric models, namely cross‐regressive (local) Spatial Lag‐X (SLX), simultaneous autoregressive (SAR) and Spatial Error (SEM) models (Anselin, 2002). Ordinary linear mixed‐effects models (Table SA4) with individual‐level and county‐level group effects justify our use of individual attributes, such as age, sex and ethnicity as the basis on which to spatially downscale discretionary time. At the same time, such models do not allow us to carry out individual‐level analysis on sub‐county geography. Moreover, segregation is a descriptive attribute of an area, such as a neighbourhood, and its impacts are more conceptually meaningful on that level, rather than a large administrative unit, like a county. Importantly, smaller geographic scale ensures that variability in local associations between discretionary time and segregation are not lost to ‘averaging out’ at a county level. Unlike conventional Ordinary Least Squares (OLS) models, our spatial models account for autocorrelation in the structure of data: SLX focuses on the local association of observed covariates, SAR emphasizes the global spatial dependence between the dependent variables, whereas SEM focuses on spatial autocorrelation in the error term. The three spatial models are complementary. For all three spatial models, we compute queen (1) contiguity weights matrices, meaning that neighbouring pairs are identified between immediately adjacent cells if they share a common border or point. Other forms of spatial contiguity or orientation (e.g., rook) may be used, there is overall no inherent reason to constrain spatial dependence structure for time availability, nor we have reasons to believe the effects would spread beyond immediate neighbours. The cross‐regressive Spatial Lag model is represented by the following equation

(5) y=Xβ+WXθ+ϵ,

where *X* is a n×k matrix of independent variables with a corresponding vector of coefficients β, WX is a cross‐regressive term obtained through multiplication of n×n matrix of weights regulating the effect of neighbouring covariates X on the time availability outcome of a cell. The SAR model is defined by the following equation

(6) y=ρWy+Xβ+ϵ,

where Wy is the autoregressive term scaled by the global spatial correlation coefficient ρ, indicating the degree of spatial dependence between the lagged dependent variable. In other words, the model predicts the impact of time availability in one cell, because of time availability patterns in surrounding regions. The SEM is shown in the following equation

(7) y=Xβ+u, whereu=λWX+ϵ,

in which u is the error term with nonspherical error variance, and λ is the error parameter. Following precedent, our model selection is based on the global Moran's *I* test for spatial autocorrelation and Lagrange multiplier tests for Spatial Lag and SEM (Anselin, 2002). Both tests indicate that our models are good candidates for explaining how ethnic segregation matters for discretionary time availability (*p* < 0.0001), considering spatial dependence between the variables. We also perform the recommended spatial Hausman test for model selection, which is designed to indicate preference for the SEM model over OLS (Pace & LeSage, 2008). The highly significant results (*p* < 0.0001) of the test indicate that neither SEM, nor OLS models are statistically sound to capture the pattern of autocorrelation in the error term. However, we include SEM and OLS models to gauge the effects of random noise on the outcome. It is instructive to observe the effects of the observed covariates on discretionary time gap in contrast to the impact of the unobserved heterogeneity on the same.

### 6.4 Control variables

In addition to the above descriptive analyses, we also examine neighbourhood‐level drivers of discretionary time availability, by taking both segregation and macro‐level sociodemographic characteristics into account. Covariates include Divergence Index (Table 3), *H* Index (Table SA5), percentage unemployed, income inequality coefficient (GINI), median household income, percentage of households headed by single women, percent of owner‐occupied housing units, percent of adults with High School diploma, and population density per square kilometer.^7^ Our covariates were derived from IPUMS, using the 2012–2016 5‐year American Community Survey attributes linked to census tracts, whose centroid (central point) falls into a particular grid cell reflecting those attributes (Oakes et al., 2018). We logarithmically transform relevant variables (% unemployed, median household income, GINI, single women‐headed households and population density) to ensure the spherical distribution of our data, residuals, and absence of multi‐collinearity. Due to data availability, our models exclude unpopulated areas. We conduct our analyses in R.

## 7 RESULTS

### 7.1 Residential segregation and the spatial variation of per capita discretionary time

**Figure 2:**
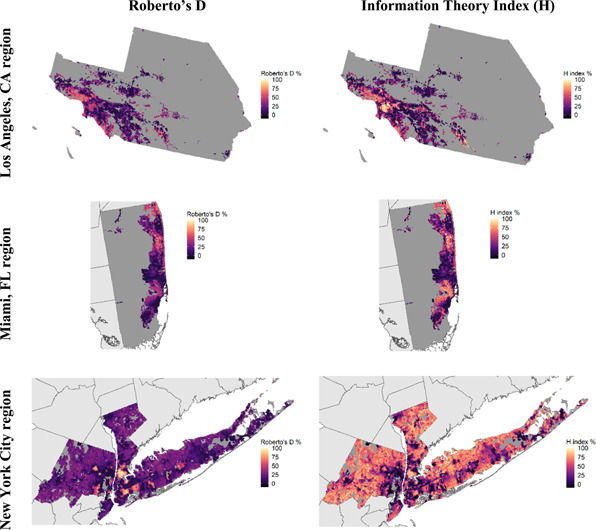
Indices of Divergence (Roberto's *D*) and Information Theory Index (H) in three regions, by 1‐km cell

**Figure 3:**
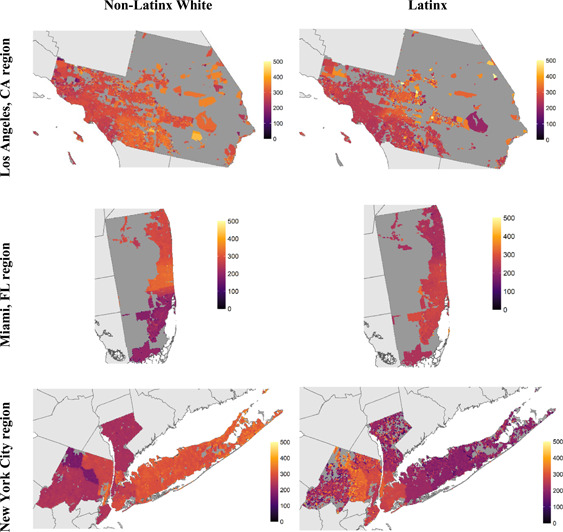
Spatial distribution of mean discretionary time in minutes per day, by 1‐km cells for Latinxs and non‐Latinx Whites

We find that the neighbourhoods located inside the urban core of both New York City and Los Angeles are highly ethnically segregated, with ample variation between neighbourhoods. Miami's urban core is less ethnically segregated than the areas immediately next to it. Figures 2 and 3 show spatial descriptive results, visually putting per capita discretionary time in conversation with ethnic segregation. Figure 2 shows the geographic distribution of ethnic residential segregation in each city's labour market commuting zone. Both segregation indices yield comparable results with greatest dissimilarity in the urban cores. Pearson correlations between D and H indices are 0.91, 0.95 and 0.68 in the LA, Miami and NYC areas. Below, we expand on the results of our Divergence Index, since it measures inequality of distribution instead of just how uneven an area is when it comes to population attributes (*H* index results are in the Supporting Information Appendix).

There is significant variation across the regions when it comes to the spatial distribution of discretionary time. Figure 3 illustrates that compared to non‐Latinx Whites, there is a sizeable disadvantage of being Latinx when it comes to discretionary time availability. Our descriptive spatial results show that there is likely a relationship between discretionary time availability and residential ethnic segregation.^8^

### 7.2 Sociodemographic characteristics and neighbourhood influences

Zooming in from the bird's‐eye‐view of the figures, Table 1 profiles 10  localities in each region. We highlight the descriptive characteristics of five localities in which Latinx have discretionary time availability advantage, and the characteristics of five localities where non‐Latinx Whites have advantage.

**Table 1:** Descriptive results of 10 locality profiles per labour market commuting zone, showing five localities in which Latinxs have an advantage when it comes to discretionary time availability, and five where non‐Latinx Whites have an advantage

Locality	Advantage	Rank	Locality	Between‐group difference in discretionary time (min/day)	Discretionary time decile	Theil's H segregation index	Poverty	GINI index	Median HH income	Unemployed adults	Owner occupied housing	Single women headed HHs	Completed High School	Population density/sq. km
New York, NY	Non‐Latinx White advantage	1	Melville, NY (South)	192.81	10	0.12	0.02	0.54	115,938	0.07	0.92	0.06	0.95	546.8
		2	Ridge, NY (North)	182.03	10	0.10	0.06	0.49	38,630	0.14	0.93	0.02	0.89	944.6
		3	Ridge, NY (Center)	177.02	10	0.10	0.07	0.48	60,474	0.05	0.78	0.16	0.91	622.1
		4	East Patchogue, NY	173.17	9	0.45	0.08	0.47	50,665	0.07	0.41	0.16	0.82	1023.4
		5	Amityville, NY	164.02	10	0.11	0.02	0.38	111,618	0.04	0.92	0.10	0.99	1498.5
	Latinx Advantage	1	Livingston, NJ (Northwest)	−181.49	5	0.13	0.02	0.48	171,125	0.04	0.96	0.05	0.96	764.7
		2	Short Hills, Millburn, NJ	−162.91	3	0.18	0.02	0.45	>250,001	0.03	0.98	0.01	0.99	636.9
		3	City of Orange, NJ (South)	−159.28	5	0.31	0.17	0.49	52,411	0.13	0.48	0.39	0.93	3766.1
		4	Livingston, NJ (Northeast)	−158.10	4	0.28	0.01	0.45	161,786	0.06	0.96	0.07	0.98	1188.0
		5	Dayton, Newark, NJ	−149.76	10	0.23	0.52	0.55	13,689	0.31	0.06	0.45	0.70	1411.2
Miami, FL	Non‐Latinx White advantage	1	Barrier Island, Ft. Lauderdale, FL	109.09	10	0.06	0.05	0.57	85,179	0.03	0.85	0.08	0.98	1381.8
		2	Cooper City, Hollywood, FL	91.70	10	0.02	0.03	0.38	121,038	0.05	0.89	0.09	0.95	1224.9
		3	Davie, FL (West)	90.06	9	0.04	0.07	0.46	85,050	0.06	0.89	0.22	0.95	689.8
		4	Boulevard Gardens, FL	89.32	10	0.46	0.31	0.37	38,351	0.13	0.62	0.46	0.75	2400.4
		5	Boca Raton, FL (Northwest)	89.31	9	0.02	0.05	0.47	87,656	0.16	0.86	0.07	0.98	505.7
	Latinx Advantage	1	Sweetwater, Miami, FL	−114.16	6	0.07	0.23	0.35	32,451	0.05	0.27	0.33	0.70	7629.3
		2	Bay Harbor Islands, FL	−110.81	3	0.00	0.13	0.55	61,324	0.07	0.45	0.19	0.94	2781.6
		3	Homestead, FL (various)	−108.80	4	0.16	0.21	0.43	50,421	0.07	0.79	0.18	0.81	166.2
		4	Tamiami, Miami, FL (North)	−108.04	7	0.06	0.13	0.44	78,004	0.12	0.89	0.15	0.83	1651.2
		5	Tamiami, Miami, FL (West)	−106.98	5	0.08	0.12	0.45	68,289	0.07	0.88	0.17	0.78	2941.4
Los Angeles, CA	Non‐Latinx White advantage	1	Sobobo Reservation, Hemet, CA	132.87	8	0.01	0.15	0.47	62,083	0.12	0.75	0.22	0.84	124.7
		2	Hemet, CA (South)	119.29	7	0.03	0.13	0.32	81,375	0.10	0.77	0.14	0.87	34.8
		3	Indio, CA (South)	118.37	8	0.13	0.12	0.57	57,199	0.09	0.69	0.13	0.84	125.1
		4	Beaumont, CA	118.33	5	0.01	0.08	0.39	72,750	0.09	0.78	0.20	0.86	28.9
		5	Murieta, CA	117.80	6	0.01	0.14	0.43	60,138	0.15	0.42	0.23	0.91	1583.9
	Latinx Advantage	1	Victorville, CA (West)	−108.72	9	0.13	0.15	0.36	59,489	0.17	0.76	0.19	0.84	199.9
		2	Ft. Irwin (vicinity)	−105.26	4	0.01	0.10	0.30	49,279	0.10	0.01	0.10	0.99	3.4
		3	Victorville, CA (vicinity)	−88.80	9	0.00	0.31	0.47	31,642	0.10	0.58	0.23	0.64	11.3
		4	Victorville, CA (vicinity)	−88.80	9	0.00	0.31	0.47	31,642	0.10	0.58	0.23	0.64	11.3
		5	Barstow, CA	−85.45	8	0.31	0.40	0.46	37,245	0.09	0.42	0.47	0.76	1425.5

As the labour market commuting zones of Miami and NYC illustrate, the areas with the most non‐Latinx White discretionary time advantage tend to be more socioeconomically advantaged. They have higher median household incomes, high home ownership rates, low levels of ethnic segregation, poverty and unemployment, and higher high school graduation rates. The areas with Latinx discretionary time advantage are interesting. Here, both Latinxs and non‐Latinx Whites are overall time poor. Yet, Latinxs in these areas have more discretionary time than non‐Latinx Whites. These localities have higher levels of poverty, lower overall household incomes, and higher levels of ethnic segregation. However, the two ends of the spectrum—areas in which Latinxs are more advantaged and where non‐Latinx Whites are advantaged when it comes to per capita daily discretionary time availability—do not differ much in terms of GINI index. This suggests that income inequality likely plays a smaller part in explaining discretionary time (dis)advantage.

The patterns found in the labour market commuting zone of LA are divergent. Here, the localities where both groups are advantaged when it comes to discretionary time tend to be more sparsely populated. Although they appear similar in terms of income inequality and segregation, these localities have lower owner‐occupied housing and lower high school graduation rates. The areas in LA where Latinxs have more discretionary time are relatively disadvantaged. This can be observed when examining median household income levels, poverty and unemployment rates.

While Table 1 is helpful for illuminating some group‐level patterns when it comes to discretionary time, segregation and sociodemographic characteristics, it does not provide an analysis of how neighbourhood and adjacent‐neighbourhood characteristics matter for segregation and discretionary time availability. We address this statistically with our regressions. Table 2 shows these results, illustrating the effects of segregation on per capita discretionary time availability, taking the above noted sociodemographic characteristics into account. The OLS model gauges the performance of the spatial models (SLX, SAR and SEM). However, on its own, it is inappropriate for prediction, as it fails to capture spatial effects by disregarding spatial autocorrelation and local context.

**Table 2:** Ordinary Least Squares (OLS), Spatial Lag – X (SLX), simultaneous autoregressive (SAR) and spatial error (SEM) models predicting per capita discretionary time availability among Latinx and non‐Latinx Whites aged 30–69, in minutes per day throughout New York City, NY, Miami, FL and Los Angeles, CA metropolitan regions

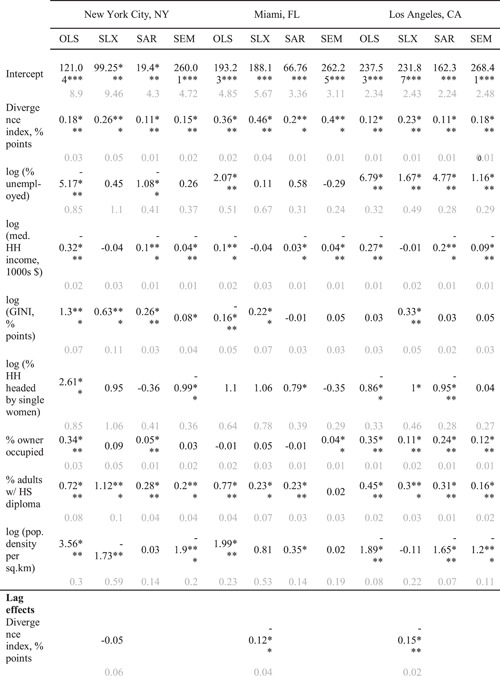
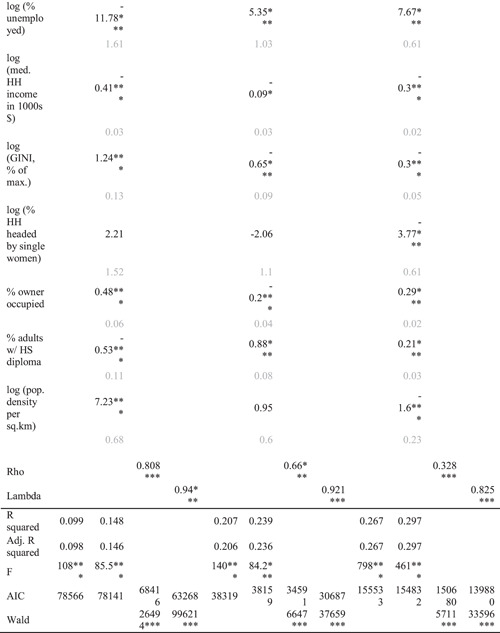

*Note*: *p < 0.05, **p < 0.01, ***p < 0.001. Standard errors are indicated in grey below.

Our SLX model measures the effects of the control variables, while incorporating adjacent neighbourhood effects on discretionary time availability. In other words, it considers both one's own neighbourhood and adjacent neighbourhoods. For example, focusing on the Divergence Index for NYC, the difference between a typical low‐segregated and high‐segregated area is 16 min of per capita discretionary time per day (assuming a 60‐percentage point difference between the two segregation states). In other words, a person from a highly segregated area has 16 more minutes of discretionary time per day than their counterpart living in a highly diverse area. Per average week, this translates to over an hour‐and‐a‐half of discretionary time. In a year, this gives us approximately 82 extra hours of discretionary time, pointing to a both substantively and statistically important difference.

Our SAR model is governed by a high degree of spatial autocorrelation (ρ>0.65). This is not surprising, given that our downscaling procedure involves adapting county‐level rates of discretionary time to the underlying cells, under the assumption of spatial uniformity. The SAR model would be more appropriate with fully representative data. Notwithstanding the preference for lag‐type model in Lagrange multiplier tests, we have no strong theoretical reasons to believe that time availability would self‐propagate across space. Thus, we show the SAR model for completeness, but do not to rely on it for predictions. Instead, we highlight our SLX and SEM models. But, as the SEM model coefficients in Tables 2 and A3 indicate for the residual effect of the segregation indices on time availability, other unobserved factors, not included in our models, may be responsible for the positive association between segregation and discretionary time availability. While we reserve judgement about SAR model impacts (Table 3 and 4A), it is likely that its global effects in the context of our segregation measures would bolster per capita time availability in neighbourhoods.

**Table 3:** Impact decomposition of the cross‐regressive spatial lag model (SLX) (top) and simultaneous autoregressive (SAR) (bottom) models for select counties of New York City, NY, Miami, FL and Los Angeles, CA metropolitan regions

	New York City, NY			Miami, FL			Los Angeles, CA		
	Direct	Indirect	Total	Direct	Indirect	Total	Direct	Indirect	Total
**SLX model impacts**									
Divergence index, % points	0.26***	−0.05	0.22***	0.46***	−0.12**	0.34***	0.23***	−0.15***	0.08***
log (% unemployed)	0.45	−11.78***	−11.33***	0.11	5.35***	5.46***	1.67***	7.68***	9.35***
log (med. HH income in 1000 s $)	−0.04	−0.41***	−0.45***	−0.04	−0.09*	−0.12***	−0.01	−0.3***	−0.31***
log (GINI, % of max.)	0.63***	1.24***	1.87***	0.22**	−0.65***	−0.43***	0.33***	−0.3***	0.03
log (% HH headed by single women)	0.95	2.21	3.16**	1.06	−2.06	−1.01	1.0*	−3.77***	−2.77***
% owner occupied	0.09	0.48***	0.56***	0.05	−0.2***	−0.14***	0.11***	0.29***	0.4***
% adults w/HS diploma	1.12***	−0.53***	0.60***	0.23**	0.88***	1.11***	0.3***	0.21***	0.51***
log (pop. density per sq. km)	−1.73**	7.23***	5.50***	0.81	0.95	1.75***	−0.11	−1.6***	−1.71***
**SAR model impacts**									
Divergence index, % points	0.15***	0.45***	0.60***	0.24***	0.35***	0.59***	0.11***	0.05***	0.16***
log (% unemployed)	−1.39**	−4.26**	−5.65**	0.69	1.02	1.70	5.02***	2.07***	7.09***
log (med. HH income in 1000 s $)	−0.12***	−0.38***	−0.5***	−0.03**	−0.05**	−0.08**	−0.21***	−0.09***	−0.29***
log (GINI, % of max.)	0.34***	1.04***	1.38***	−0.01	−0.02	−0.03	0.03	0.01	0.04
log (% HH headed by single women)	−0.46	−1.42	−1.88	0.94	1.39	2.33	−1.0***	−0.41***	−1.41***
% owner occupied	0.07***	0.21***	0.27***	−0.01	−0.02	−0.03	0.25***	0.10***	0.36***
% adults w/HS diploma	0.36***	1.1***	1.46***	0.27***	0.4***	0.66***	0.33***	0.13***	0.46***
log (pop. density per sq. km)	0.04	0.14	0.18	0.41*	0.61*	1.02*	−1.74***	−0.71***	−2.45***

* *p* < 0.05.

** *p* < 0.01.

*** *p* < 0.001.

Table 2 shows compares significant results of the SEM model with the SLX model, accounting for the spatial correlation of residuals (errors), measuring spillover and containment effects due to variables not captured by our models. We find that in all models and all regions, residential ethnic segregation is statistically significant for discretionary time availability. For example, continuing our focus on the segregation index for NYC, but looking at our SEM results, we find that one percentage point increase in segregation (D) increases per capita daily discretionary time availability by 0.15 min. This takes the unobserved effects in consideration. This is best interpreted in conjunction with the total effect of the SLX model (Table 3), since the SLX model indicates the effects captured locally with variables. In this case, the comparison suggests that the unobserved heterogeneity in covariates diminishes the effect of residential segregation on discretionary time availability in the NYC region. Residential segregation (Divergence Index) increases discretionary time availability in all regions, but its effects are uneven. For example, in the Miami, region, Latinxs lose 0.2 min relative to non‐Latinx Whites per percentage point increase in segregation (results not shown).

Examining sociodemographic characteristics (percentage of households headed by single women, percentage of owner‐occupied households, percentage of adults with a high school diploma, population density), we find that they are significant when it comes to the relationship between ethnic segregation and discretionary time availability. Together, they explain over one‐quarter of the variance in the association between discretionary time availability in the Miami area. The results for the New York City and Los Angeles regions offer comparable findings.

Table 3 and A6 decompose the contextual impacts of the explanatory variables in the SLX model. Direct effects refer to the specific neighbourhood. Indirect effects refer to the effects of adjacent neighbourhoods. Total effects are the sum of the direct and indirect effects for a given neighbourhood. If we deem low and moderate segregation to be about 25 percentage points apart on the scale of either the D or H index, then in the NYC region, this translates into an increase in per capita discretionary time availability between low and medium segregation neighbourhoods by 0.22×25=5.5 min/day (nearly 34 h/year) for the D index and by 0.19×25=4.75 min/day for the H index. Similarly, holding other predictors constant, in the Miami and LA regions, the D index predicts 8.5 and 2 min increase in discretionary time, while the H index predicts a 3‐min increase in the Miami region, with no difference in the LA region.

As Table 3 shows, in most instances indirect effects are significant, whereas the direct ones are not. However, this is not the case for the segregation measures. This suggests that time availability in a neighbourhood is significantly and substantially influenced by the sociodemographic characteristics of the neighbourhood, more so than by the characteristics of the adjacent neighbourhoods. The degree of segregation in adjacent neighbourhoods, along with their socioeconomic environments, are also significant, but are less substantial predictors of discretionary time availability at a given location. For example, if we examine the indirect columns for NYC's segregation indices (Table 3 and A4), we find that segregation in surrounding neighbourhoods has no effect on per capita discretionary time availability in the neighbourhood under examination. However, it increases discretionary time availability by 0.25–0.26 min per percentage point increase of either index within the neighbourhood. Though generally smaller in magnitude than direct effects, both segregation indices in adjacent neighbourhoods predict negative spillover effect on time availability in a given neighbourhood, ranging from 0.12 to 0.2‐min reduction per point increase in segregation in the Miami and LA regions. In other words, increasing segregation in the surrounding neighbourhoods decreases time availability in one's own neighbourhood.

## 8 DISCUSSION

This study systematically links ethnic residential segregation and per capita discretionary time availability, an understudied yet highly responsive measure to sociodemographic opportunities and constraints. Our main findings are: (1) In most areas of NYC, LA, and Miami, Latinx individuals have less discretionary time than non‐Latinx White individuals. (2) In most neighbourhoods, segregation is correlated with having more per capita discretionary time. (3) However, increasing segregation in surrounding neighbourhoods decreases time availability in one's own neighbourhood. (4) Neighbourhood‐level sociodemographic characteristics shape the relationship between segregation and discretionary time.

Incorporating research from the neighbourhood effects literature, literature on Latinx segregation, and from the sociology of time, our study highlights the need to reorient current theorizing toward also including sociotemporal inequalities. We show that both within‐neighbourhood and adjacent‐neighbourhood influences affect the needed resource of discretionary time. However, the situation is more complex than the existing literature suggests. Unlike when it comes to strictly economic outcomes, in most neighbourhoods, segregation is overall beneficial when it comes to per capita discretionary time availability. This suggests that in addition to socioeconomic, cultural and well‐being benefits, ethnic enclaves may also impart temporal advantages (Cutler et al., 2008; Edin et al., 2003; Fine, 2002; Fischer & Massey, 2000). It is likely that in addition to distributing economic risks, the presence of extended kinship and close‐knit peer networks in ethnically segregated communities translate to time‐saving and time‐sharing benefits (Menjivar, 1997).

Yet, increasing segregation in the surrounding neighbourhoods decreases per capita time availability in one's own neighbourhood. In other words, one may have an advantage when it comes to per capita time availability in a segregated neighbourhood, but this advantage diminishes when the surrounding neighbourhoods are also segregated. This suggests that there may be diminishing marginal returns with increasing segregation in the surrounding areas. There are multiple mechanisms through which this could operate. For example, in a scenario where one's own neighbourhood is not segregated—but is surrounded by highly segregated neighbourhoods—an individual could lose discretionary time while navigating the disadvantages select segregated neighbourhoods contend with (Alba & Foner, 2015, Crowder & South, 2008). This is a plausible spillover effect on per capita discretionary time. In the second scenario, where one's neighbourhood is highly segregated, just like the surrounding neighbourhoods, an individual will still lose discretionary time. This could happen in multiple ways: if one's neighbourhood is segregated along the same ethnic dimensions (e.g., Own‐Latinx—surrounding—Latinx and own‐White, surrounding—White) or different ethnic dimensions (eg. Own‐Latinx—Surrounding‐White or vice versa). In the first scenario, the same explanation as before may stand (incongruence of resource distributions and exchanges). In the second scenario, we could speculate about possible competition for finite resources or the potential spatial dilution of resources. Different economic and sociodemographic mechanisms likely operate in each scenario. Future work also needs to consider how individual and structural discrimination may play a role in the above scenarios. This line of theorizing presents a rich avenue for further research.

We show that neighbourhood‐level sociodemographic characteristics shape the relationship between residential ethnic segregation and time availability. They are responsible for approximately 25% of the variance between ethnic segregation and per capita time availability. However, they are highly context‐dependent. For example, single women‐headed households tend to have more discretionary time in NYC, but have less discretionary time in LA. This highlights the necessity for future studies on how contextual social safety nets shape time availability. Further, income inequality as measured by the GINI index is also context‐dependent when it comes to per capita discretionary time availability. This points to the need for more research (deploying causal analyses) specifically testing the mechanisms between segregation and time availability^9^. This study needs to pay careful attention to the ways in which the resource of time differs from income.

As the unobserved heterogeneity in our SEM models indicates, other variables—unmeasured socioeconomic, sociodemographic and cultural factors—also matter. To unpack this, future data collection efforts could incorporate how living arrangements, household characteristics, peer network characteristics, along with differing labour market experiences may influence the relationship between discretionary time and ethnic segregation. This study should pay particular attention to how socioeconomic and neighbourhood‐level factors shape the prevalent cultural discourses surrounding time availability.

Due to data availability considerations, we could only include three large, coastal, U.S.‐based labour market commuting zones. Thus, it is unclear if ethnic segregation plays the same role in other contexts and for other ethnic and/or racial groups when it comes to per capita time availability. This would be worthwhile to examine in more rural regions of the U.S. and in other countries. This requires fine‐scale, individual level, geographically linked microdata (currently unavailable in surveys incorporating both time use and segregation measures). Future research needs to ground this area of inquiry with ethnographic studies exploring how discretionary time itself may be conceptualized differently by racial and ethnic groups inhabiting differing regions of the globe (Williams et al., 2016). From the methodological standpoint, further extensions to our algorithms are possible. With greater availability of spatial time use data, it would be possible to include additional factors (e.g., income or household structure), from which to compute estimates for downscaling. Likewise, improvements in spatial accuracy could be achieved with a yet more nuanced post‐downscaling adjustment that includes as its smoothing weight a composite index of individual or area‐level variables that could impact the availability of discretionary time. Lastly, future research needs to explore alternative spatial models and specifications with additional areal covariates that could provide superior explanatory power.

Researchers need to revisit the issue of gender when it comes to how segregation affects time availability. As our unit of analysis is the 1 km grid, we focus on how macro‐level, contextual factors matter, relying on spatial modelling to understand the geographic distribution of segregation and time availability. We include the effect of percentage of households headed by single women (a socioeconomic disadvantage indicator), but this is not the same as fully tackling gender differences when it comes to how ethnic segregation shapes time availability.^10^ Knowing the existing differences in discretionary time availability between the genders (Goodin et al., 2008), there is a possibility that ethnic segregation may matter for women's time availability differently. This is a highly policy‐relevant topic that needs more nuanced examination.

Our understanding of how ethnic residential segregation influences life chances remains incomplete without an examination of how it matters for sociotemporal inequalities. Our study contributes to the current literature in several ways. To our knowledge, no prior studies have put neighbourhood and adjacent‐neighbourhood effects in conversation with how ethnic segregation may shape the necessary resource of discretionary time. Our findings highlight that is worthwhile to put prevalent theoretical perspectives from the neighbourhood effects literature in conversation with theories on ethnic residential segregation and from the sociology of time. By doing so, we illustrate that it is imperative to consider discretionary time as a resource when theorizing about how ethnic segregation influences other resources. We specifically focus on honouring the experiences of the Latinx population, as considering their population size, their discretionary time availability profiles are vital for shaping the overall time use profile of the U.S. (Bernstein, 2013; Lichter et al., 2015). Our work serves as an important first step in tracing how temporal processes are connected to processes of stratification.

## Supporting information

Supporting information.

## Footnotes

1
https://www.ers.usda.gov/data-products/commuting-zones-and-labor-market-areas/

2
https://www.census.gov/quickfacts/fact/note/US/RHI725219, since the Latinx census‐demarcated category also includes individuals who have roots in Latin America but may be Black, White, Asian, and so forth, we limit our analyses to Latinx versus non‐Latinx White.

3 See Table SA2 for detailed list of counties and sample sizes.

4 See Tables SA1–A3 in the Supporting Information Appendix for group‐specific estimates, standard errors and sample sizes for each county.

5 See the Supporting Information Appendix for an extensive description of our downscaling and adjustment procedures.

6
https://www2.census.gov/programs-surveys/demo/about/housing-patterns/multigroup_entropy.pdf

7 As we measure ethnic segregation via our segregation index, to avoid collinearity and artificial inflation of variance in coefficients, we do not disaggregate our controls by ethnicity.

8 See Figures SA1 and SA2 for differences in mean discretionary time (min/day/1‐km cells).

9 This is outside the scope of this paper, and we were also restricted by data limitations.

10 In Table SA1 of the Supporting Information Appendix we show the mean daily discretionary time availability in hours per day for non‐Latinx Whites and Latinx, by age group, sex and county of residence. As expected, women have overall less discretionary time.
