# Supplementary material for: Tell me who's your neighbour and I'll tell you how much time you've got: The spatiotemporal consequences of residential segregation
Source: Popul Space Place. 2022 Mar 28;28(7):e2561. doi: 10.1002/psp.2561 (PMC9787190; doi:10.1002/psp.2561)
Supplement: Supplementary file 1 — Supporting information. [file PSP-28-e2561-s001.docx]

**Tell Me Who's Your Neighbor and I’ll Tell You How Much Time You've Got: The Spatiotemporal Consequences of Residential Segregation**

**Online Appendices**

A technical note on our smoothing algorithm

Equation 1 shows the formal mathematical expression for our smoothing algorithm, estimating the smoothed value of discretionary time at each cell location $t_{i}^{*}$.

$$t_{i}^{*} = \mathbb{I}(dist(i, b_{i}) \leq w_{c_{i}}) [t_{i} - exp(\frac{-k_{i}d_{i}}{10})(t_{i} - \overline{t}_{b_{i}}) ] + \mathbb{I} (dist(i,b_{i}) > w_{c_{i}})t_{i} \boldsymbol{(1.1)}$$

$$w_{c_{i}} = \sqrt{A_{c_{i}}} \boldsymbol{(1.2)}$$

where $\overline{t}_{b_{i}}$ is the mean discretionary time computed at the point on the border $b_{i}$, closest to the grid cell $i$, while $t_{i}$ is the value of discretionary time for cell $i$. $w_{c_{i}}$ is the radius of the circular buffer, dependent on the size of the county $c$ that encompasses cell $i$. The buffer is used to compute the zonal mean value of discretionary time at each point on the border $b_{i}$, situated at distance ${dist}_{i}$ in kilometers from the centroid of its corresponding grid cell. $k_{i}$ is the smoothing sensitivity coefficient, where$k \in[0,1]$, adjusting the smoothing power so that the value of the coefficient increases proportionally with the fraction of urban population within a given cell, ensuring that urban locations retain more weight than rural ones. This is an important consideration, since values of discretionary time are numerically shaped more by residents of urban places, as compared to the residents of the rural areas. Note that $t_{i} = t_{i}^{*}$ in cases where any given cell is located far from the closest county border, beyond the threshold distance where smoothing comes into effect. The threshold distance $w_{c_{i}}$ is determined by the square root of the area $A$ of county in which any given cell is located. This ensures that smaller counties will not be oversmoothed, as compared to larger ones.

| \| **Figure A1.** Overall availability of discretionary time for Non-Latinx Whites and Latinx, 1-km cells by decile. \| \| \| --- \| --- \| \| **Los Angeles, CA region** \| 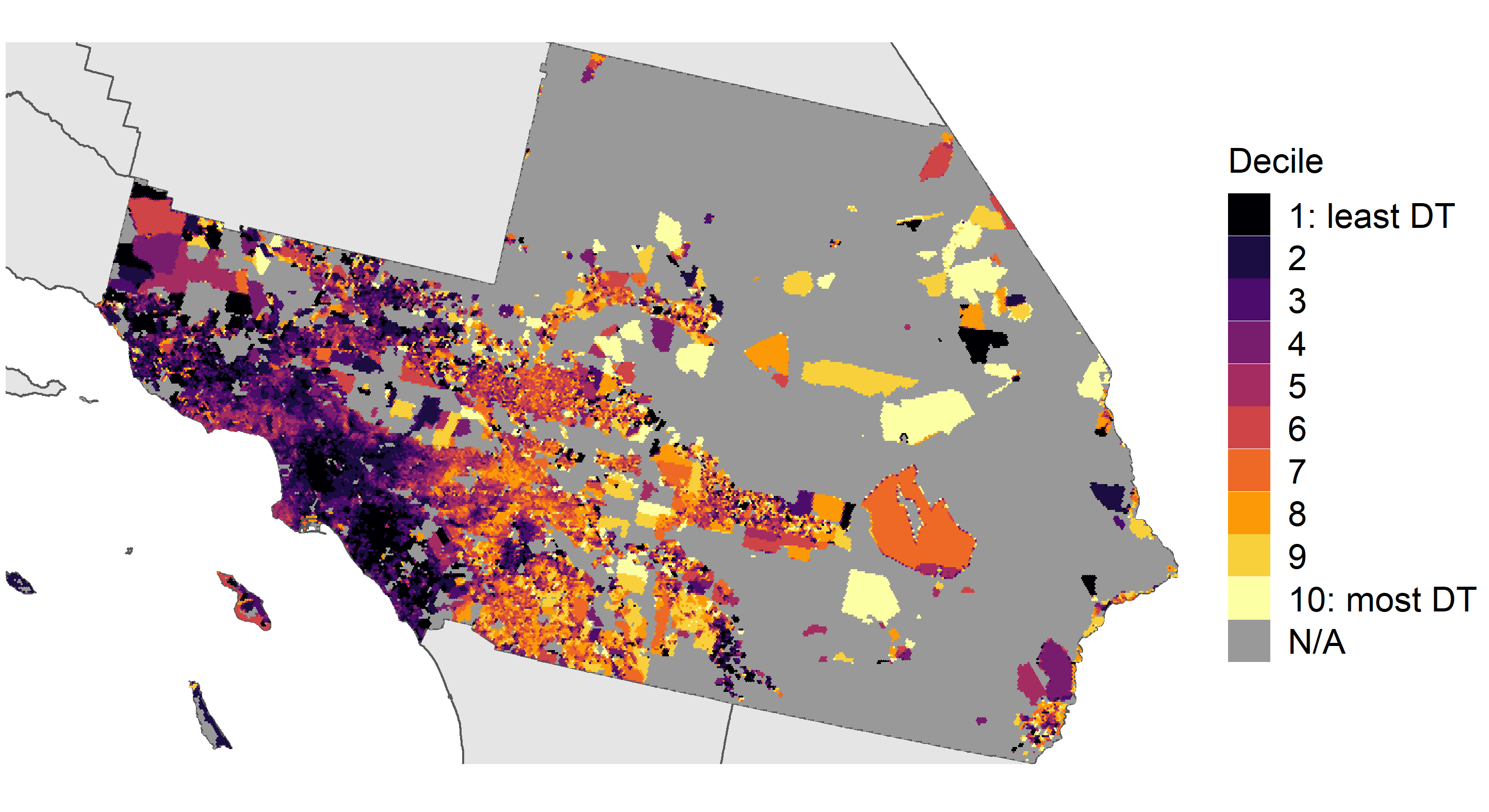 \| \| \| **Miami, FL region** \| 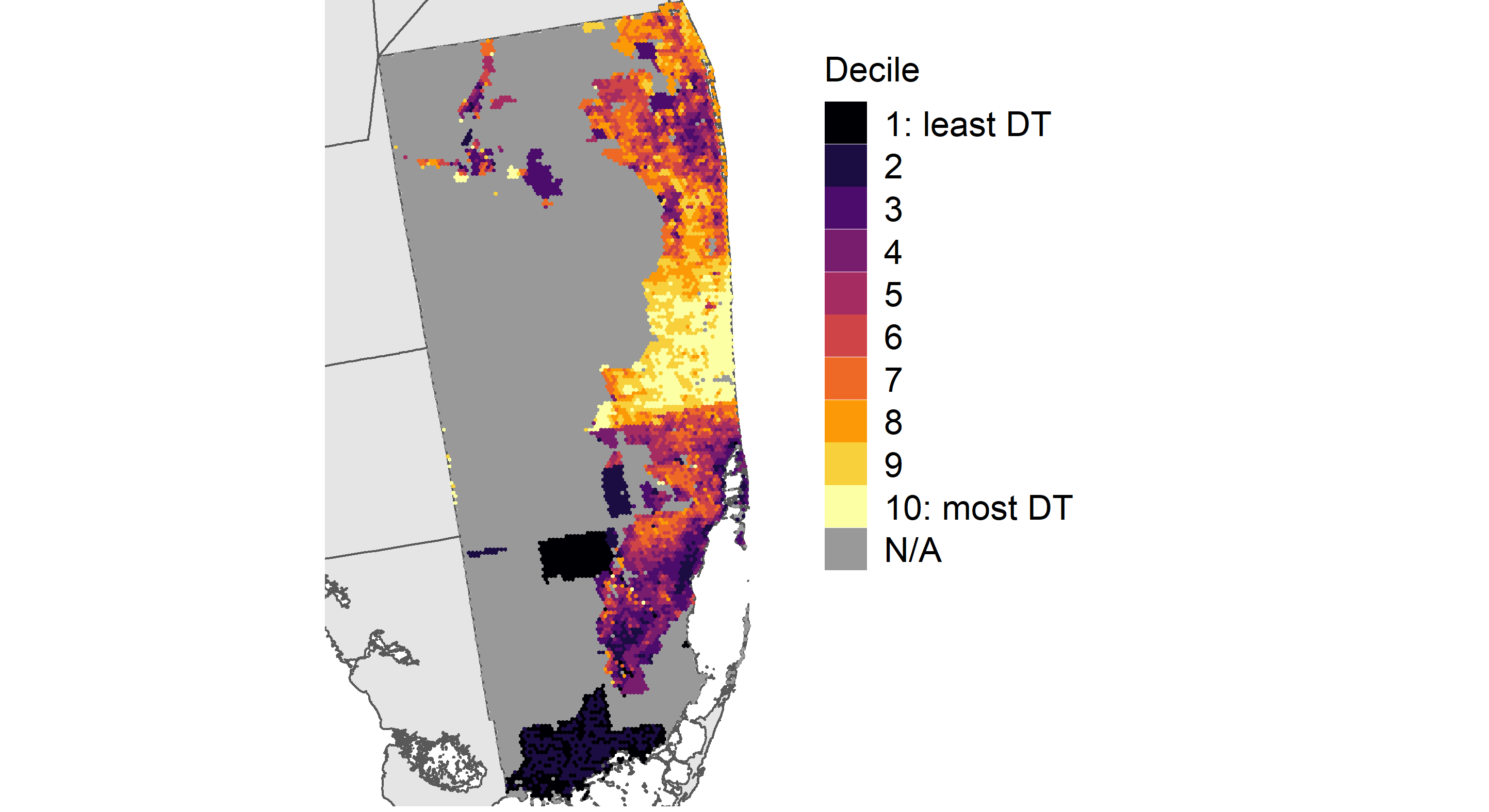 \| \| \| **New York City region** \| **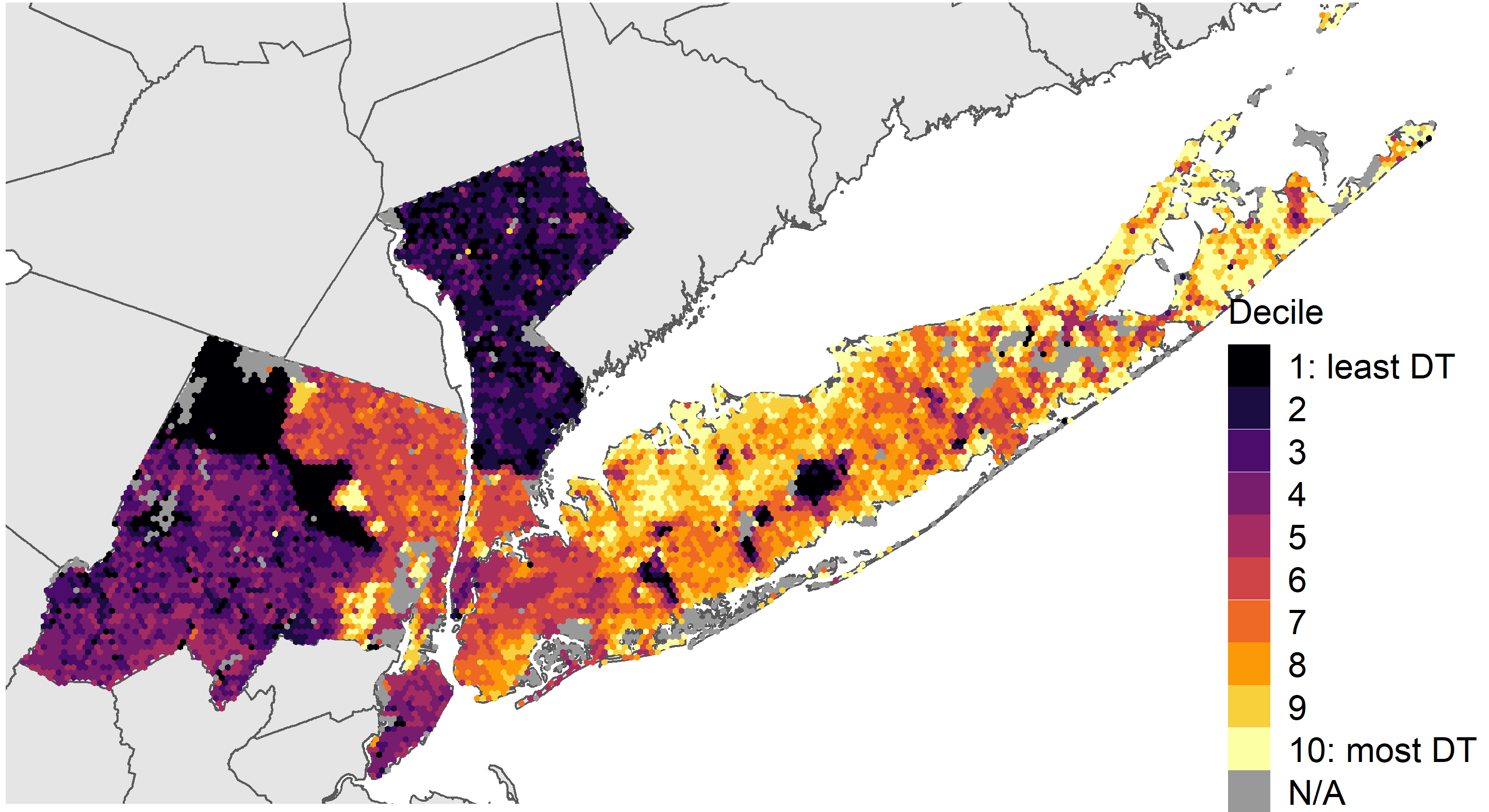** \| \|   **Figure A2.** Difference in mean discretionary time in minutes per day by 1-km cells between Non-Latinx Whites and Latinx. | | |
| --- | --- | --- | --- | --- | --- | --- | --- | --- | --- | --- | --- | --- | --- |
|  | **NL White – Latinx difference in min./day** | **Regional distribution by**  **ethno-racial group** |
| **Los Angeles, CA region** | 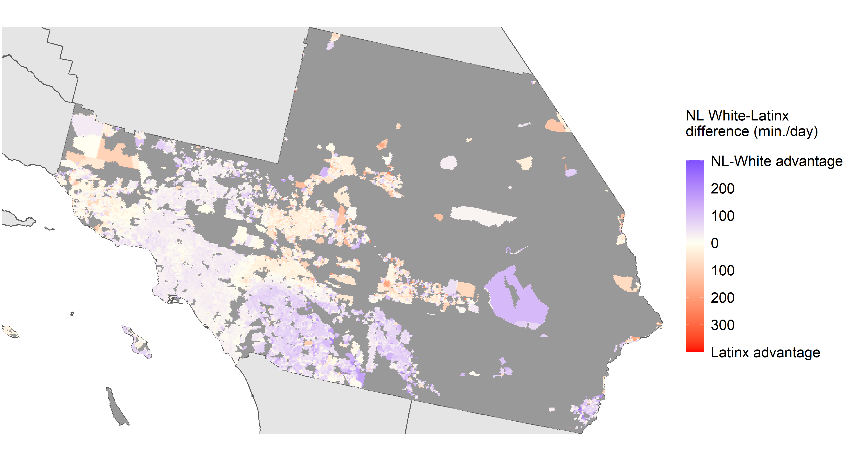 | 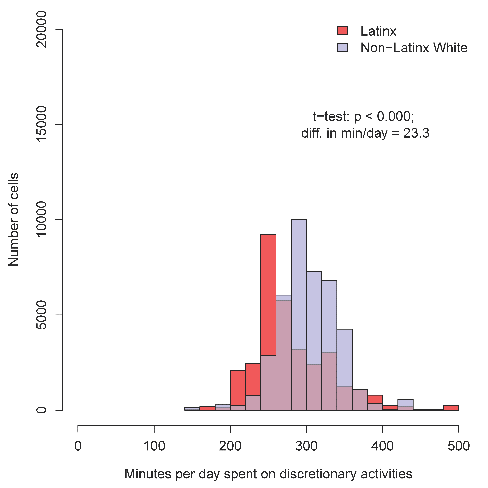 |
| **Miami, FL region** | 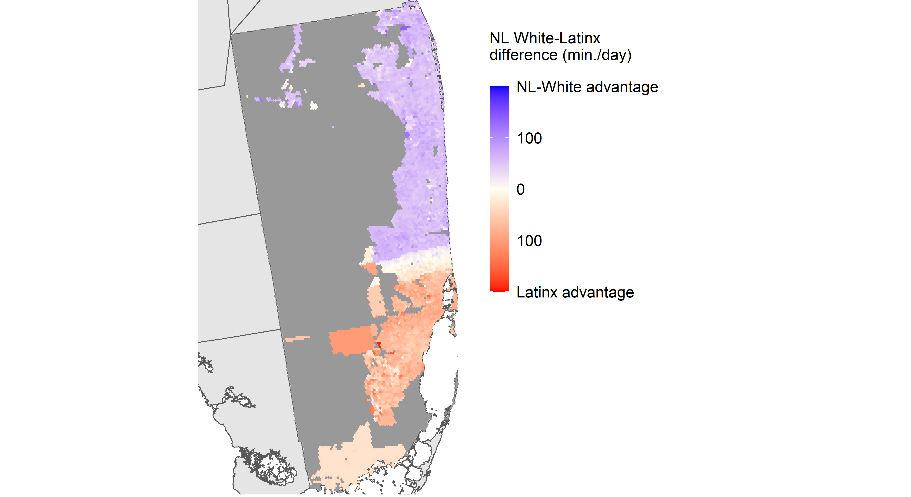 | 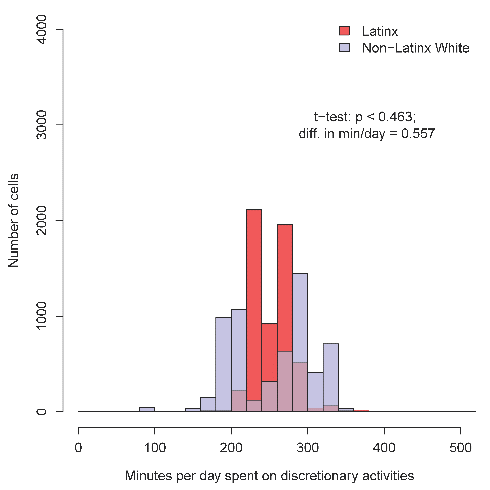 |
| **New York City region** | **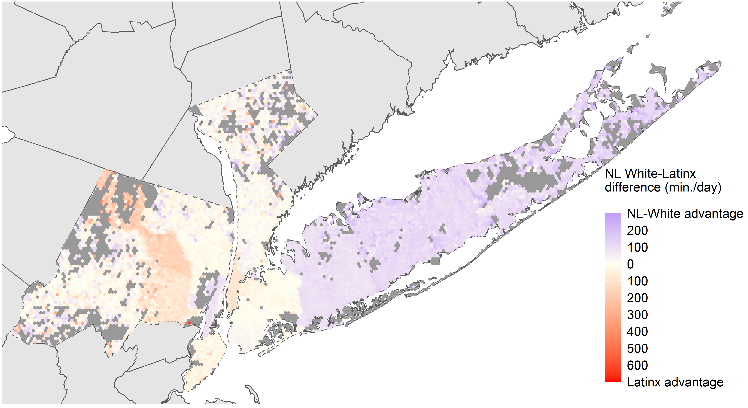** | 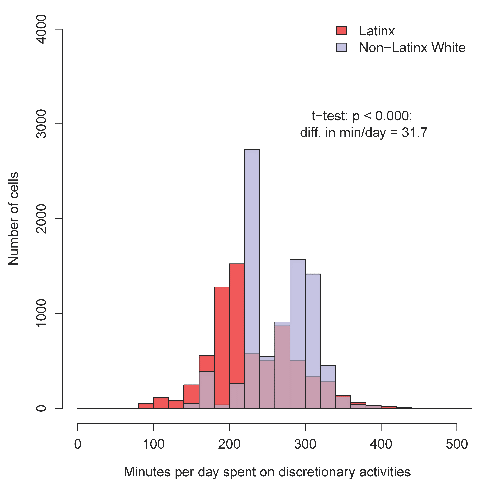 |
